# Multilevel associations between prostate cancer testing and socioeconomic position: a population-based register study from Stockholm, Sweden

**DOI:** 10.1136/bmjph-2025-003493

**Published:** 2026-02-17

**Authors:** Balram Rai, Marta Rado, Anna Sara Oberg, Ralf Kuja-Halkola, Mark S Clements

**Affiliations:** 1 Department of Medical Epidemiology and Biostatistics, Karolinska Institutet, Stockholm, Sweden

**Keywords:** Public Health, Social Medicine, Sociodemographic Factors

## Abstract

**Introduction:**

Prostate cancer testing is associated with both individual and area-level socioeconomic position (SEP), but the multilevel nature of this association is unclear and contribution of SEP to the spatial variation is unknown. This study investigated the association of widespread opportunistic prostate-specific antigen (PSA) testing with SEP measures and quantified the extent to which multilevel measures of SEP contributed to the observed spatial variation in PSA testing.

**Methods:**

A population-based register study was conducted, encompassing 471 335 men aged 40 years and older without a prior prostate cancer diagnosis residing in the Stockholm region in 2016. We used hierarchical Bayesian logistic regression models with spatial random effects to estimate the associations between PSA testing and SEP measures.

**Results:**

Men aged 70–79 in the highest income quartile had the highest proportion (35.2%, 95% CI 34.5% to 35.9%) of PSA testing in 2016. Adjusting for age and spatial variation, men with at least 12 years of education for having a PSA test had a 22% (95% CI 19% to 25%) higher odds compared with men with less than 9 years of education. For small area level variance in PSA testing, the highest proportion (42.0%) explained was seen for income.

**Conclusions:**

The findings suggest a moderate association between opportunistic prostate cancer testing and SEP measures at the individual and area levels. The SEP measures at the individual and area levels substantially explained the spatial variation in PSA testing, where income was the strongest driver. Future strategies for prostate cancer testing should be aware of SEP differentials at both individual and area level to reduce socioeconomic inequities in incidence and mortality.

**WHAT IS ALREADY KNOWN ON THIS TOPIC**

- Prostate-specific antigen (PSA) testing for prostate cancer screening is controversial but still commonly used. It has previously shown that PSA testing varies by individual-level socioeconomic position (SEP) and by area-level SEP, and that PSA testing exhibits substantial spatial heterogeneity.

**WHAT THIS STUDY ADDS**

- This study analyses multilevel associations between prostate cancer testing and SEP and quantifies the proportion of spatial variation in PSA testing explained by cross-level measures of SEP. We found moderate associations between individual-level SEP and prostate cancer testing even after adjusting for area-level SEP and spatial variation.
- SEP at both levels, particularly income at area level, explains a substantial proportion of the spatial variation in PSA testing.

**HOW THIS STUDY MIGHT AFFECT RESEARCH, PRACTICE OR POLICY**

- Methodologically, our findings support the use of multilevel models to describe cross-level associations between prostate cancer testing and SEP. In particular, we found evidence that SEP operates at both the area level and the individual level for prostate cancer testing.
- SEP was found to explain a substantial proportion of the spatial heterogeneity, suggesting that screening interventions could target low-income areas.
- Differences in prostate cancer testing by SEP are expected to lead to differences in costs, benefits and harms for prostate cancer.
- In planning for organised prostate cancer testing in European countries, it is important to understand the past and current socioeconomic differentials. There is increasing evidence that organised testing could lead to substantial differences in participation by SEP, which highlights the significant policy challenges to address the socioeconomic gradients in testing to achieve more equitable prostate cancer outcomes.

## Introduction

 Prostate cancer is the second most common cancer worldwide after lung cancer, and the most common cancer in Europe among men.^1^ In Sweden, around 10 000 new prostate cancer cases are diagnosed every year, and around 2500 deaths are attributed to prostate cancer.^2^ In 2022, the 10-year prevalence for prostate cancer in Sweden was 1637 per 100 000 males.^3^ Screening for cancer is one of the primary methods for early detection in a population. The prostate-specific antigen (PSA) test is the most common test used for early detection and management of prostate cancer. Using the PSA test for screening has both potential harms and benefits.^4 5^ PSA testing is associated with reduced prostate cancer mortality but with a risk of overdiagnosis.^6 7^ The PSA test is not highly specific to detect prostate cancer, as the PSA levels could be elevated due to causes other than prostate cancer, such as benign prostatic hyperplasia and subclinical prostatic inflammation.^8^

In 2018, the Swedish National Board of Health and Welfare recommended against population-based PSA screening for prostate cancer, which aligned with previous recommendations against PSA-based screening including the US Preventive Service Task Force.^9^ Despite these recommendations and uncertainty around PSA testing, PSA testing has been very common in the Stockholm region.^10^ The European Council recently recommended that member states consider a stepwise approach for organised prostate cancer testing (OPT).^11^ In 2022, the Swedish Social Department recommended that health regions investigate possible approaches to OPT. Following this recommendation, most of the regions in Sweden, including the Stockholm region, have introduced OPT pilot studies using PSA in combination with MRI, where men are informed about the potential harms and benefits of testing.^12^

Men with lower individual-level socioeconomic position (SEP) are at an increased risk of prostate cancer mortality, mostly due to diagnostic delay and poorer prognosis.^13^ At an area level, international reviews have found substantial geographical variation in prostate cancer outcomes, with lower SEP areas having lower prostate cancer incidence and higher mortality rates.^14 15^ In Sweden, an association of neighbourhood deprivation with prostate cancer incidence and mortality suggests the possibility of contextual effects on these outcomes.^16 17^ Socioeconomic disparities in prostate cancer incidence and mortality could be partially attributed to differences in the uptake of PSA testing at both an individual and an area level. Therefore, this highlights the importance of describing and understanding the socioeconomic disparities in testing to reduce the disparities in incidence and mortality.

Individual-level associations of PSA testing with multiple measures of SEP have been explored in previous studies.^18^,^20^ Studies have also focused on the association of PSA testing using specific individual-level measures of SEP, such as education,^21 22^ income^23^ and ethnicity.^24 25^ A handful of studies have explored associations between PSA testing and area-level SEP.^26 27^ Studies on health equity account for multilevel measures of SEP preferably using a hierarchical/multilevel model^28^; however, such evidence is lacking for the associations between prostate cancer testing and SEP. Furthermore, few studies have explored the spatial heterogeneity in prostate cancer testing,^29 30^ and little is known as to the extent that individual-level or area-level SEP explain the spatial heterogeneity.

The aims of this study were to investigate multilevel associations of SEP with opportunistic prostate cancer testing and to examine the proportion of spatial variance which was explained by SEP. Specifically, we quantified the proportion of spatial variance explained by different measures of SEP; income, education, country of birth and civil status. We hypothesised that (a) there would be moderate associations between opportunistic prostate cancer testing and individual-level SEP even after controlling for area-level SEP and (b) measures of individual and area-level SEP would substantially explain any spatial variation in testing.

## Data and methods

### Data sources

Data on PSA testing were obtained from the Stockholm PSA and Biopsy Register which collects data from the three laboratories that analyse PSA tests performed in the Stockholm region. The three laboratories are Karolinska University Laboratory (KUL), Aleris and Unilabs, who analyse all PSA tests done in the Stockholm region. KUL analyses around 60% of all PSA tests performed, followed by 20% each by Aleris and Unilabs.

These data were further linked to health and population registers for information on date of birth, area of residence, internal (in/out of Stockholm region within Sweden) and external migration (in/out of Sweden), cancer diagnosis and measures of SEP, using the unique identification number assigned to all residents in Sweden. The population for this study was men aged 40 years and above without a prior prostate cancer diagnosis and living in the Stockholm region in 2016.

### Small areas

We used SAMS (Small Area Marketing Statistics) as the small areas, a regional classification system provided by Statistics Sweden which identifies small areas in Sweden. The areas were defined based on digital boundaries by Statistics Sweden, which used a method that was similar to that used for postal codes. There were 9200 SAMS areas in Sweden, including 890 areas in the Stockholm region. These areas had been previously used to analyse the neighbourhood or area-level effects on prostate cancer incidence^17^ and mortality.^16^

### Socioeconomic position

SEP is a construct to identify the socioeconomic conditions of individuals and areas which affect health outcomes^31^ including cancer.^32^ SEP can be measured through different measures at the individual level (compositional) and the area level (contextual).^28^ The SEP measures used in this study were country of birth, education, income and civil status extracted from the STATIV database, which is a longitudinal database for integration studies from Statistics Sweden. The choice of SEP measures was based on previous empirical evidence on the associations with prostate cancer testing and availability of data.

Country of birth was categorised as Nordic countries (Sweden, Denmark, Finland, Norway and Iceland), Europe except Nordics and outside Europe. We grouped the Nordic countries because we expected their health systems to be similar and hence also their healthcare seeking behaviour. Education was expressed in terms of completed educational length in years, categorised into: short (less than or equal to 9 years); medium (>9 and ≤12 years); and long (>12 years). The categorisation for education was based on Swedish education nomenclature which is called Svensk utbildningsklassifikation. This classification had been aligned with the international classification ISCED 97 (International Standard Classification of Education). Income was represented as the individual annual disposable income (income after tax deduction). Due to a strong association between income and age, we created age-specific income quartiles by 10-year age groups (age groups 40–49, 50–59, 60–69, 70–79, 80+), where Q1 corresponded to lowest income quartile and Q4 to highest income quartile. We included civil status under a broad definition for SEP based on earlier evidence on an association with testing.^33^ Civil status was categorised as (1) never married or registered in a partnership, (2) separated/divorced and (3) married or registered in a partnership. Area-level SEP was measured as the proportion of men in each category of these measures at the small area level (eg, the proportion of income quartile Q1 in an area, and the proportion of men with lower education in an area).

### Statistical analysis

We reported the proportion of men without a prior prostate cancer diagnosis having at least one PSA test in 2016 by all the measures of SEP and by 10-year age-groups (40–49, 50–59, 60–69, 70–79, 80+). Bayesian logistic regression was used to model the proportion of men having a PSA test. The individual-level associations of PSA testing with the measures of SEP were estimated using ORs with 95% CI. For the main findings, the ORs for SEP were conditional on age and marginal for unmodelled covariates. In a sensitivity analysis, the ORs were conditional on age and other SEP measures and were marginal over the unmodelled covariates. We estimated the proportion of spatial variance explained in PSA testing by different measures of SEP. The proportion of spatial variance was calculated in three steps: (a) including SEP at the individual level; (b) including SEP at the small area level and (c) including both individual and area-level SEP simultaneously.

### Spatial model

The traditional BYM (Besag, York and Mollie) model used for spatial disease mapping does not allow us to independently interpret the spatially structured component (local or spatially correlated) of variance and the unstructured component (global or independent random) due to confounding and scale dependence. We used a reparameterisation of the BYM model which allowed for an easy and scale-independent interpretation of the hyperparameters.^34^ Further, we used some penalised complexity priors to avoid overfitting, by penalising the model for deviations from the base model (no spatial structure) until there was enough support for a complex model, especially in small areas with limited data.^35^ Let $Y_{ij}$ represent a binary outcome for a man indexed by j within area i takes a PSA test following a Bernoulli distribution denoted by $Y_{ij}∼Bern\left(P_{ij}\right),$where $P_{ij}$ represents the probability for a man in area i to have a PSA test in the study population. We then modelled for this probability using a logistic function


$$logit\left(P_{ij}\right)=\beta_{1}^{T}x_{ij}+\beta_{2}^{T}x_{i}+s_{i}+u_{i}$$

where $\beta_{1}$ and $\beta_{2}$ were the vectors of coefficients for covariates at the individual-level and the area-level, $s_{i}$ was the spatially structured component of the random effect, and $u_{i}$ was the unstructured global random effects, which were independent between areas. The random effects were reparametrised as


$$b_{i}=\frac{1}{\sqrt{\tau }}\left[\sqrt{1-\phi }\;s_{i}+\sqrt{\phi }\;u_{i}\right]$$

where 0≤ϕ≤1 was the mixing parameter suggesting the attributable fraction to spatial component of the random effects (for example, ϕ = 0 suggests no spatial component), and where τ represented the total precision (inverse of total variance).


$$s_{i}∼CAR\left(W\right),\;\;var\left(s_{i}\right)=1$$

The $s_{i}`s$ were modelled using a scaled conditional autoregressive distribution with spatial weight matrix W. The spatial weight matrix W was constructed using queen’s contiguity, where each area borrows strength from all the other areas with which it shares any boundary.^36^ Queen contiguity was preferred over other approaches to avoid the restrictive interaction in rook contiguity approach and choosing arbitrary threshold distance for distance-based approach.


$$u_{i}∼N\left(0,1\right),\;\;var\left(u_{i}\right)=1$$

The $u_{i}`s$ were modelled as independently and identically distributed standard normal distribution. Since both structured and unstructured components of random effects are scaled to variance 1,


$$var\left(b_{i}\right)=\frac{1}{\tau }$$

The $var\left(b_{i}\right)$ represents the total variance of structured and non-structured random effects.

The covariates included an intercept term, a factor variable adjusting for 10-year age groups (with a reference group of 40–49 years), and various measures of SEP. We interpreted the exp(β) as ORs with 95% credible interval (CI). The base model (without SEP) included only age groups as covariates and then different measures of SEP were subsequently added to the model (with SEP) and the proportion of variance explained was estimated for each SEP measure.

The proportion of variance explained in PSA testing by a given measure of SEP was estimated using


$$Proportion\;of\;variance\;explained=1-\frac{{\left[\;var\left(b_{i}\right)\right]}_{with\;SEP}}{{\left[var\left(b_{i}\right)\right]}_{without\;SEP}}$$

where ${\left[var\left(b_{i}\right)\right]}_{withoutSEP}$ was extracted from the model adjusted only for age-groups and ${\left[var(b_{i})\right]}_{withSEP}$ was extracted from the model when SEP measure was included in the model. The posterior convergence was checked by inspecting the marginal posterior distributions to ensure the stability of the parameter estimates for fixed effects and random effects parameters. For sensitivity analyses, we plotted marginal posteriors of fixed effects and random effects parameters including structured and unstructured random effects with and without all the measures of SEP at the small area level and municipality level. We also plotted the complete density plots for variances with and without all the measures of SEP at both levels. Finally, we plotted the density plot for variances for (a) individual-level SEP, (b) area-level SEP and (c) combining individual and area-level SEP. We used the R-INLA package^37^ to fit these models and all the statistical analyses were performed using R software V.4.2.2.

## Results

**Figure 1:**
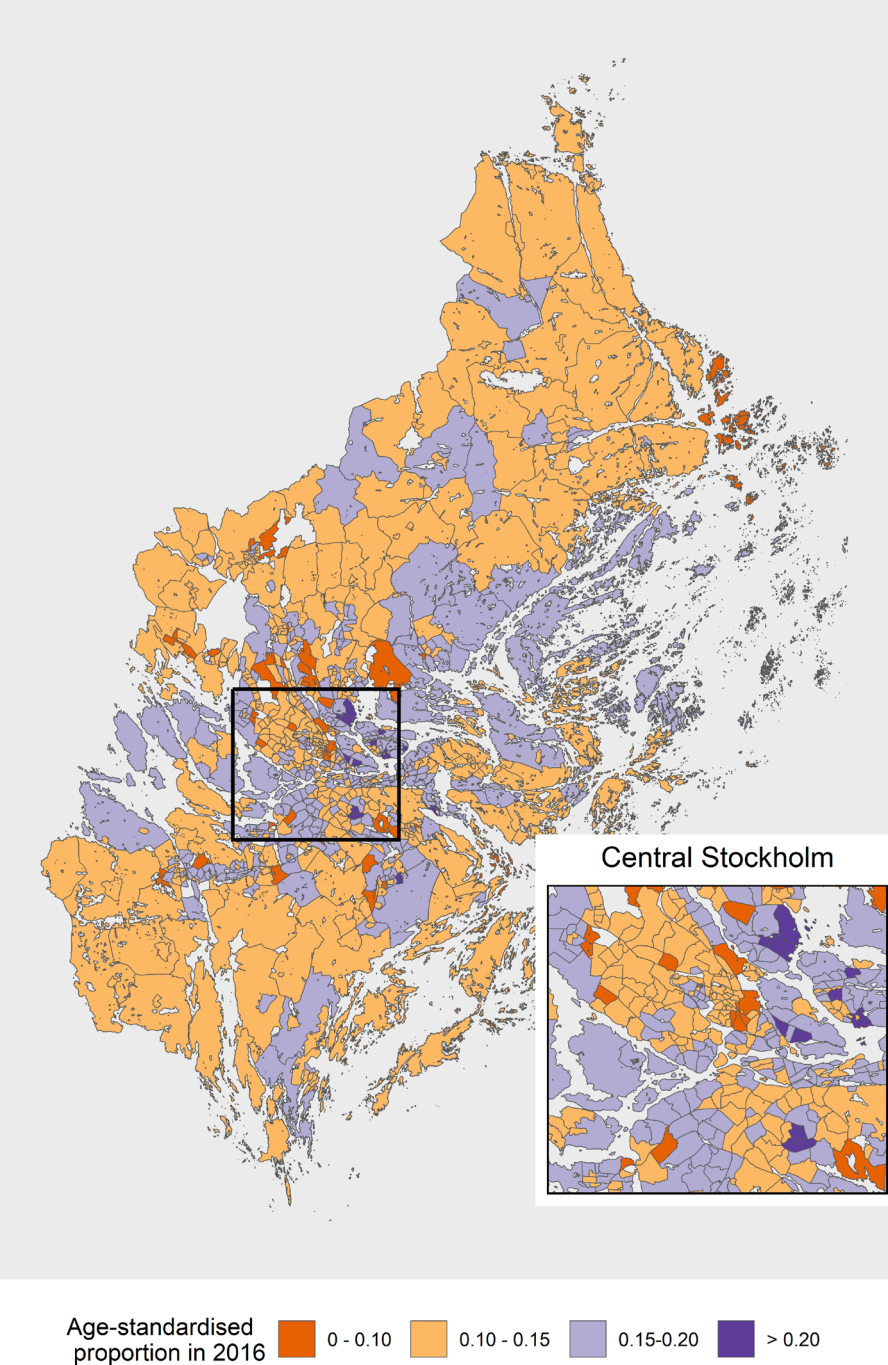
Age-standardised proportion of men having a PSA test by small area in the Stockholm region, 2016. PSA, prostate-specific antigen.

There were 4 71 335 men aged 40 years and over living in Stockholm in 2016, with no prior record of prostate cancer. Of those men, 71 061 (15.1%, 95% CI 15.0% to 15.2%) had at least one PSA test in 2016 (table 1). The proportion of men having a PSA test increased by age through to 70–79 years and then decreased. Men aged 70–79 in the highest income quartile had the highest proportion (35.2%, 95% CI 34.5% to 35.9%) of PSA testing. We found that men who were born in the Nordic countries had the highest level of PSA testing among all age groups. Men with more years of education tended to have higher proportions of PSA testing, as did men with higher incomes. There was a marked spatial variation in men taking a PSA test in the small areas in 2016 (figure 1).

**Table 1:** Proportion of men having at least one PSA test by measures of socioeconomic position (SEP) and 10-year age groups in the Stockholm region, 2016

SEP	40–49	50–59	60–69	70–79	80+	Total
	N (%)	N (%)	N (%)	N (%)	N (%)	N (%)
Country of birth						
Outside Europe	20 733 (4.5)	17 081 (12.4)	9579 (19.5)	2915 (23.4)	756 (20.9)	51 064 (11.3)
Europe except Nordics	14 738 (4.2)	11 154 (11.7)	7932 (20.6)	5488 (25.6)	2190 (21.6)	41 502 (13.1)
Nordics	115 451 (5.1)	104 264 (14.4)	78 730 (22.2)	56 728 (28.3)	23 414 (23.2)	378 586 (15.8)
Education						
Short (less than 9 years)	14 894 (4.7)	18 802 (12.1)	18 038 (18.2)	15 089 (23.7)	7833 (20.3)	74 655 (15.3)
Medium (9–12 years)	60 460 (5.0)	56 539 (14.1)	38 810 (21.0)	26 625 (27.5)	10 563 (22.9)	192 997 (15.0)
Long (more than 12 years)	75 857 (4.9)	57 273 (14.3)	39 614 (24.1)	23 440 (30.9)	7961 (25.8)	204 145 (15.1)
Income						
Q1 (lowest quartile)	37 368 (3.8)	33 206 (9.7)	24 180 (16.1)	16 287 (21.6)	6572 (19.4)	117 614 (11.3)
Q2	37 510 (4.9)	32 969 (13.1)	24 153 (20.2)	16 294 (25.2)	6589 (21.9)	117 516 (14.1)
Q3	37 740 (5.0)	33 169 (15.4)	24 056 (23.3)	16 289 (29.4)	6571 (23.1)	117 825 (16.0)
Q4 (highest quartile)	37 623 (6.1)	33 080 (17.5)	24 099 (27.4)	16 273 (35.2)	6572 (27.8)	117 646 (18.9)
Civil status						
Never married	54 698 (4.3)	39 036 (11.2)	20 175 (16.6)	7907 (20.5)	1581 (18.6)	123 396 (9.7)
Separated/divorced	17 019 (5.2)	25 217 (13.8)	22 036 (19.7)	16 647 (25.2)	9842 (20.2)	90 761 (16.4)
Married/registered in partnership	79 302 (5.3)	68 290 (15.5)	54 245 (24.5)	40 543 (30.4)	14 886 (25.4)	257 266 (17.2)

N represents the population and % represents the proportion of men having a PSA test in each group.

PSA, prostate-specific antigen.

**Figure 2:**
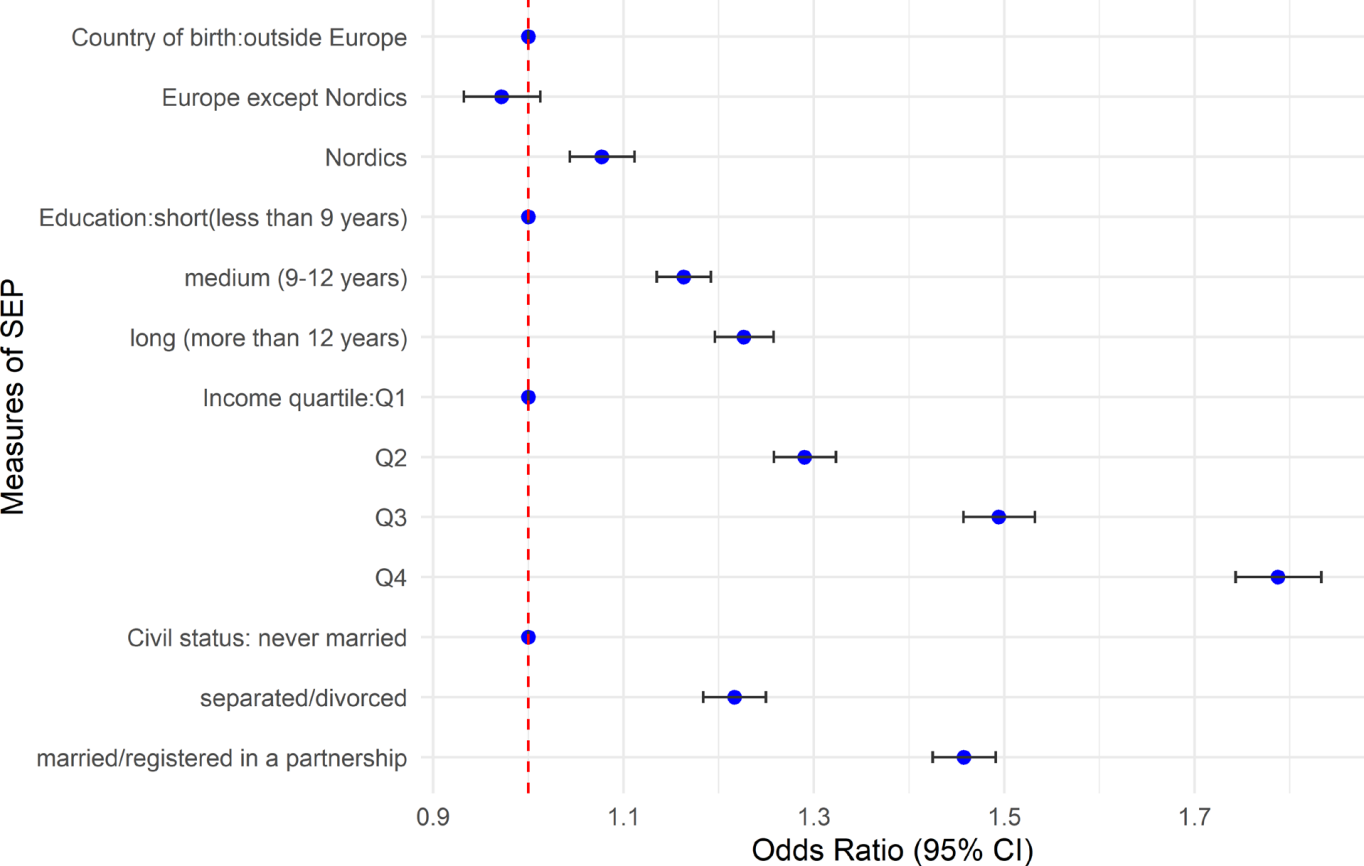
Age-adjusted ORs for associations of PSA testing with measures of individual-level socioeconomic position (SEP) in the Stockholm region, 2016. PSA, prostate-specific antigen.

Adjusting for age and accounting for spatial variation, men with 12 or more years of education had 22% higher odds (95% CI 19% to 25%) for having a PSA test compared with men with less than 9 years of education (figure 2). Men in higher income quartiles had greater odds of having a PSA test (Q4: 78% (95% CI 74% to 83%), Q3: 49% (95% CI 45% to 53%), Q2: 29% (95 % CI 25% to 32%)) compared with men in the lowest income quartile. Married men or men registered in a partnership had a 45% (95% CI 42% to 49%) higher odds of having a PSA test compared with those never married. Men born in the Nordic countries had a 7% (95% CI 4% to 11%) higher odds of having a PSA test compared with men born outside Europe. The associations remained significantly positive with a slight reduction in the estimates for income and civil status when adjusted for other measures of SEP. When we included all the SEP measures, country of birth was not significantly associated with PSA testing (online supplemental figure S1).

Of the different types of SEP measures, income had the highest proportion of spatial variance explained, both when evaluated at the individual (32.3%) and the small area level (42.0%; table 2). After income, civil status had the highest proportion of spatial variance explained at the individual level (14.3%) and area level (38.4%) respectively. Across all types of SEP measures, the area-level explained a higher proportion of spatial variance compared with the individual-level. Combining the different types of SEP measures, approximately half of the spatial variance was explained by area-level measures, whereas individual-level measures explained 37.5% (table 2). There were limited gains in the proportion of spatial variance explained if we included SEP measures at individual and area-level simultaneously compared with area-level alone, which was also checked with the density plots for variances in these models (online supplemental figure S8). However, the models including both individual and area-level SEP had better model fits compared with individual or area-level SEP alone. The results for fixed effects from these models are presented in online supplemental table S1.

**Table 2:** Proportion of spatial variance explained by measures of SEP at individual and area level in the Stockholm region, 2016

Measures of SEP	Model specification for SEP at different levels					
	Individual level		Area level		Individual+area level	
	Proportion	Model fit	Proportion	Model fit	Proportion	Model fit
Income	32.4	−183 923	42.0	−184 864	41.8	−183 894
Education	9.1	−184 885	28.5	−184 912	28.7	−184 824
Country of birth	4.0	−184 983	18.4	−184 945	18.3	−184 942
Civil status	14.3	−184 432	38.5	−184 876	37.9	−184 358
All measures	37.5	−183 577	49.5	−184 859	49.5	−183 562

Proportions reported as percentages of variance explained, and model fits as marginal log-likelihoods.

SEP, socioeconomic position.

We found that our models converged and estimates from the models were stable (online supplemental figure S2). The marginal posteriors for structured random effects were narrower compared with unstructured effects at the small area level (online supplemental figures S3 and S4) and municipality level (online supplemental figures S5 and S6). There was a significant difference between the variances for models with and without SEP measures at small area level (online supplemental figure S7), whereas the difference was not significant at municipality level (online supplemental figure S8). There was a significant difference between the variances for models with and without SEP and including SEP at individual and area level (online supplemental figure S9). Comparing the summary measures for random effects at small area and municipality level, the uncertainty in the summary estimates was much higher at municipality level (online supplemental table S3) compared with small area level (online supplemental table S2). The computation time was around 1–2 min for a single model for a sample size of around 500 000 individuals and 890 small areas for random effects. For more details about the sensitivity analyses, please refer to the online supplemental information.

## Discussion

In summary, we found moderate associations between opportunistic prostate cancer testing and SEP at the individual level and area level. The different measures of SEP partially explained the spatial variation in opportunistic prostate cancer testing at the small area level. Income had the strongest association with testing among all the measures of SEP, including individual-level association and in terms of proportion of variance explained. All other measures of SEP, including education, civil status and country of birth, were also associated with prostate cancer testing at both the individual level and area level. Our findings for the associations of PSA testing with SEP are consistent with previous literature at the individual level^18 19 33 38^ and area level.^26 30^ To the best of our knowledge, this study is the first to examine both the cross-level associations between SEP and prostate cancer testing and to describe the extent to which SEP accounts for the spatial variation in PSA testing.

There has been some debate on how to model for compositional and contextual effects on a health outcome. Jen *et al* argued that area-level analyses could be affected by the ecological fallacy and they emphasised the role of compositional variables in accounting for spatial variation.^39^ We assessed the contribution of individual-level and area-level SEP measures towards the explained spatial variance, including the combination of both, and found that area-level SEP measures explained much of the broader contextual effects. The individual-level SEP measures improved the model fit in terms of marginal log likelihoods but did not add to the explained spatial variance when already accounting for area-level SEP measures. There could still be some unmeasured contextual factors that could contribute to the unexplained heterogeneity after SEP such as access to general practitioner services,^40 41^ lack of awareness or public health campaigning.^42 43^

The association of SEP with prostate cancer testing is multifaceted, with potential for negative consequences at both ends of the distribution of SEP. First, higher levels of opportunistic testing in the higher SEP groups have been found associated with an increased incidence of low-grade or insignificant cancers,^44^ leading to overdiagnosis and overtreatment. Second, lower participation in prostate cancer testing among lower SEP groups has been linked with delayed diagnosis^13^ and a higher likelihood of metastatic diagnosis,^45^ both of which lead to poorer prognosis. The frequent testing among the older age groups (70+) is also concerning as it may increase the risk of overdiagnosis and overtreatment^46^ given the lower chances of aggressive prostate cancer or prostate cancer-specific death for those with low PSA values.^47^ There has not been sufficient evidence from screening trials to understand potential harms and benefits of PSA testing for older age groups.

As a strength of this study, the analysis was based on a population-based individual linkage of PSA tests with cancer registration and men spatially encoded for small homogeneous areas. The granularity in the spatial data allowed us to capture more variation in SEP which would be masked at higher geographical levels such as municipality. We used an individual-level model with spatial random effects to adjust for individual-level and area-level SEP measures simultaneously rather than an ecological approach which accounts only for area-level SEP.

As a limitation, we did not have access to more recent small area data to evaluate the association of SEP with organised testing due to institutional restrictions. We also lacked access to primary care data which could be used to explore other area-level factors including the geographical distribution of clinics offering PSA tests which could contribute to unexplained spatial heterogeneity in prostate cancer testing. As a further limitation, we lacked some measures of wealth, including other sources of income (eg, stocks and home-ownership), which could further contribute to unexplained spatial heterogeneity. We did not handle multiple testing among men to avoid the overdispersion and chose any testing as an indicator, but the average number of tests among men was 1.35 (SD=0.82) and median being 1 (IQR: (1,1)). In this study, we have focused on SEP for a given time period instead of a temporal component. A spatio-temporal analysis of SEP is certainly interesting but would be methodologically complicated.

For future research, it would be useful to reproduce these findings in other populations. While the level of testing may vary between populations, we hypothesise that the cross-level associations and the proportion of variance explained will be similar in other European populations. There has been a growing literature on geo-spatial approaches for cancer prevention, and to understand the geography of inequalities in cancer outcomes.^48^ Further research is needed to explore the other factors that could better explain the spatial heterogeneity in prostate cancer testing. Based on preliminary evidence from a pilot OPT programme in Sweden, the socioeconomic gradients of participation were worsened in the OPT setting compared with an unorganised setting.^33^ Since the OPT pilot studies are in an implementation phase, they highlight the policy challenges to address the socioeconomic disparities and the spatial variation for the structured testing in all the groups. There have been substantial recent changes in prostate cancer management, including the introduction of MRI, the use of other reflex diagnostic tests such as STHLM3, initiating pilot studies to investigate the feasibility of OPT and the introduction of new treatments which could affect prostate cancer survival. A key policy question is to assess whether these changes will lead to reduced socioeconomic disparities in prostate cancer testing and outcomes. A future evaluation of an OPT programme will require data on prostate cancer outcomes by SEP at both the small area level and at the individual level to monitor whether the inequalities are exacerbated.

## Supplementary material

10.1136/bmjph-2025-003493 online supplemental file 1

## Data Availability

Data may be obtained from a third party and are not publicly available.
