# Supplementary material for: Multilevel associations between prostate cancer testing and socioeconomic position: a population-based register study from Stockholm, Sweden
Source: BMJ Public Health. 2026 Feb 17;4(1):e003493. doi: 10.1136/bmjph-2025-003493 (PMC12927357; doi:10.1136/bmjph-2025-003493)
Supplement: online supplemental file 1 [file bmjph-4-1-s001.docx]

**Supplementary table and figures**

In summary, we performed extensive sensitivity analysis to check the convergence of the models, stability of parameters including fixed-effects and random effects parameters, cross-level interactions of individual- and area level measures of SEP. We found that our models converged and estimates from the models were stable.

The adjusted odds ratio (adjusted for age and other measures of SEP) suggested a slight reduction in effects of income and civil status on PSA testing, but the effects of country of birth became insignificant (Figure S1). Adjusting for age, there were minimal reductions in the effects of individual level measures of SEP after including the area-level measures in the model (Table S1). We also tested for the convergence of our models by checking the marginal posterior distributions for fixed effect parameters in terms of log-odds ratio (Figure S2), random effects parameters including precision and the mixing parameter $\phi$ (Table S2). The marginal posterior distributions for fixed effects parameters were stable and unimodal. As part of additional sensitivity analyses, we also plotted posterior distributions for variances of random effects in the null model (including only age) and including all individual-level measures of SEP in the model (Figure S7) and area-level measures (Figure S9). These plots showed significant reduction in the variance for model with SEP compared to model without SEP. We also distinguished structured and unstructured random effects by plotting the marginal posteriors for random effects of model without SEP (Figure S3) and model with SEP (Figure S4). As expected, the structured random effects have lower variances compared to unstructured part as it represents the spatial random effects (local) and borrows strength from neighbouring areas. We performed multicollinearity diagnostic tests and computed generalised variance inflation factors (VIFs) for all the measures of SEP. The VIF values ranged from 1.01 to 1.05, which did not pose marked multicollinearity concerns.

To highlight the importance of performing analysis on small-area spatial heterogeneity, we extended the sensitivity analyses by using municipalities as the spatial unit. There were larger uncertainties in random effects and parameters (Table S3) and insignificant difference in the variance for model with SEP compared to model without SEP (Figure S8).

As a post hoc analysis, we have now tested for nonlinearity of age using a random walk smoothing function in INLA. Using a non-linear age, the revised model minimally affects the fixed effect parameters in terms of log odds ratio (see the table below for example of income) and random effect parameters in terms of the variance explained

| *Income categories* | *using 10-year age-groups* | *using smoothing function for age* |
| --- | --- | --- |
| *Lowest quartile (Q1)* | *ref.* | *ref.* |
| *Q2* | *0.255* | *0.256* |
| *Q3* | *0.402* | *0.416* |
| *Q4* | *0.581* | *0.598* |

Figure S1. Age-adjusted odds ratios for associations of PSA testing with all the measures of individual-level socio-economic position (SEP) in one model (adjusted) compared with each separately in a model (unadjusted) in the Stockholm region, 2016


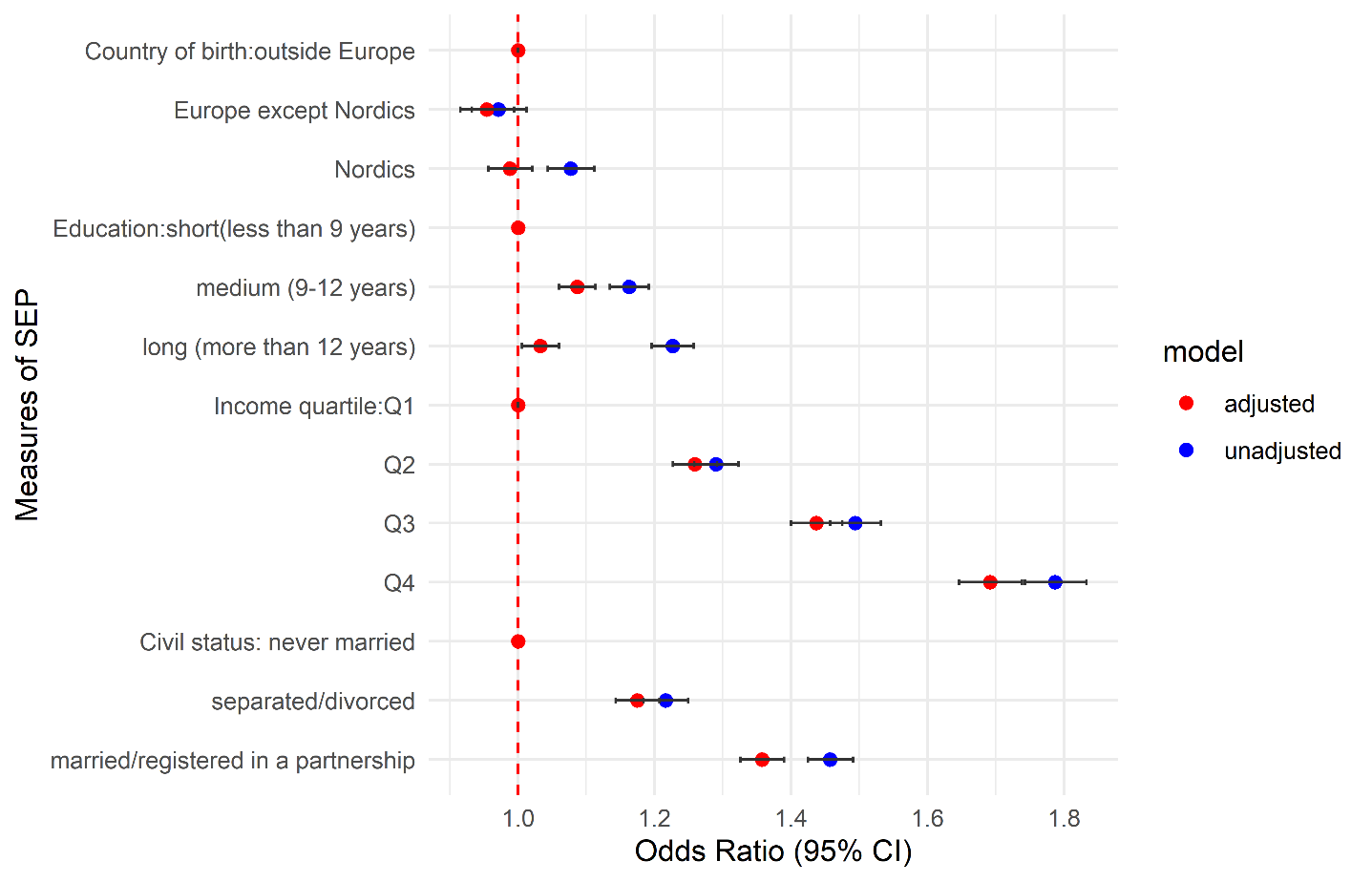


Table S1. Association of PSA testing individual-level and area-level SEP measures in the Stockholm region, 2016. Regression coefficients, i.e. log (odds ratios) from separate models per SEP measure.

| **Measures** | **Individual level** | **Area level** | **Individual+ Area level** | |
| --- | --- | --- | --- | --- |
|  |  |  | **Individual** | **Area** |
| **Income (quartiles)** |  |  |  |  |
| Q1 | Ref. | Ref. | Ref. | Ref. |
| Q2 | 0.255 (0.230, 0.280) | 0.620 (0.197, 1.044) | 0.251 (0.225, 0.276) | 0.381 (-0.044, 0.806) |
| Q3 | 0.402 (0.377, 0.427) | 0.465 (0.198, 0.732) | 0.390 (0.365, 0.416) | 0.095 (-0.173, 0.363) |
| Q4 | 0.581 (0.555, 0.606) | 1.236 (1.008, 1.463) | 0.559 (0.533, 0.584) | 0.705 (0.475, 0.934) |
| **Education** |  |  |  |  |
| Short | Ref. | Ref. | Ref. | Ref. |
| Medium | 0.151 (0.126, 0.175) | 0.988 (0.562, 1.414) | 0.142 (0.118, 0.167) | 0.852 (0.425, 1.278) |
| Long | 0.204 (0.178, 0.229) | 1.326 (1.075, 1.576) | 0.184 (0.159, 0.209) | 1.145 (0.894, 1.397) |
| **Country of birth** |  |  |  |  |
| Outside Europe | Ref. | Ref. | Ref. | Ref. |
| Europe except Nordics | -0.029 (-0.070, 0.012) | 0.988 (0.221, 1.756) | -0.039 (-0.080, 0.002) | 1.029 (0.261, 1.798) |
| Nordics | 0.075 (0.044, 0.107) | 1.036 (0.744, 1.328) | 0.050 (0.018, 0.082) | 0.933 (0.699, 1.286) |
| **Civil status** |  |  |  |  |
| Never married | Ref. | Ref. | Ref. | Ref. |
| Separated/divorced | 0.196 (0.169, 0.223) | -1.076 (-1.548, -0.601) | 0.195 (0.168, 0.222) | -1.173 (-1.647, -0.696) |
| Married/registered in partnership | 0.376 (0.354, 0.399) | 0.725 (0.504, 0.948) | 0.364 (0.341, 0.387) | 0.422 (0.199, 0.647) |

Table S2. Summary measures for random effects (precision and $\rho$) at the small area level for all the models using different measures of individual-level SEP

|  | **Precision (1/variance)** | | | **Spatial component (**$\boldsymbol{\phi}$**)** | | |
| --- | --- | --- | --- | --- | --- | --- |
| **Models** | **Estimate** | **95% CI** | | **Estimate** | **95% CI** | |
| Without SEP | 19.754 | 17.097 | 22.760 | 0.131 | 0.035 | 0.280 |
| COB | 20.507 | 17.704 | 23.691 | 0.137 | 0.037 | 0.288 |
| Education | 21.681 | 18.705 | 25.056 | 0.129 | 0.034 | 0.277 |
| Income | 28.910 | 24.580 | 33.813 | 0.160 | 0.050 | 0.323 |
| Civil status | 23.050 | 19.828 | 26.710 | 0.146 | 0.043 | 0.300 |
| All measures | 31.442 | 26.626 | 36.962 | 0.173 | 0.058 | 0.342 |

Table S3. Summary measures for random effects (precision and $\rho$) at the municipality level for all the models using different measures of individual-level SEP

|  | **Precision (1/variance)** | | | **Spatial component (**$\boldsymbol{\phi}$**)** | | |
| --- | --- | --- | --- | --- | --- | --- |
| **Models** | **Estimate** | **95% CI** | | **Estimate** | **95% CI** | |
| Without SEP | 59.492 | 34.003 | 95.320 | 0.625 | 0.145 | 0.969 |
| COB | 60.340 | 34.447 | 96.580 | 0.630 | 0.148 | 0.970 |
| Education | 67.476 | 38.126 | 108.628 | 0.579 | 0.117 | 0.957 |
| Income | 58.881 | 30.052 | 101.299 | 0.589 | 0.101 | 0.968 |
| Civil status | 66.219 | 37.540 | 106.620 | 0.639 | 0.160 | 0.970 |
| All measures | 76.626 | 42.831 | 124.651 | 0.577 | 0.121 | 0.953 |

Figure S2. Posterior density for fixed effect parameters for the model including age and income as covariates


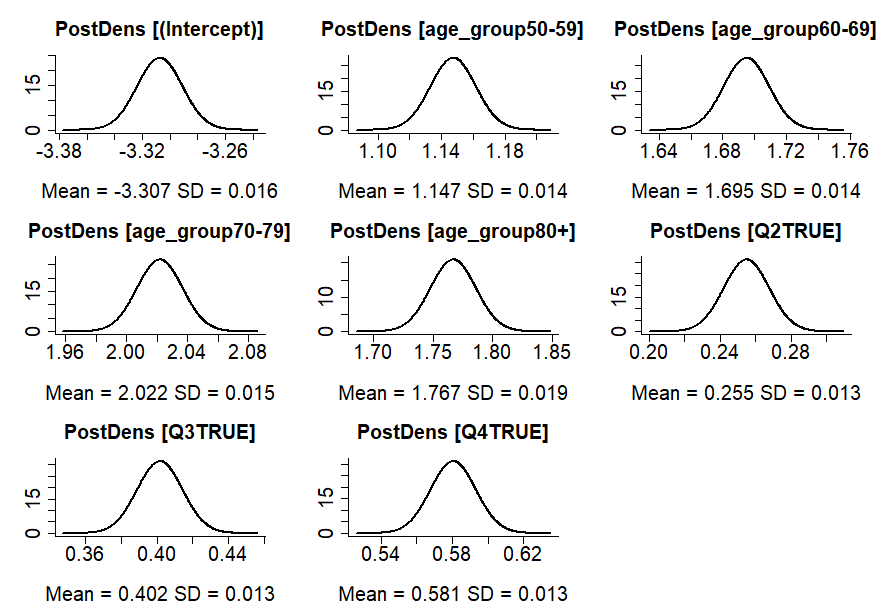


Figure S3. Marginal posteriors of random effects in the model without any measures of SEP


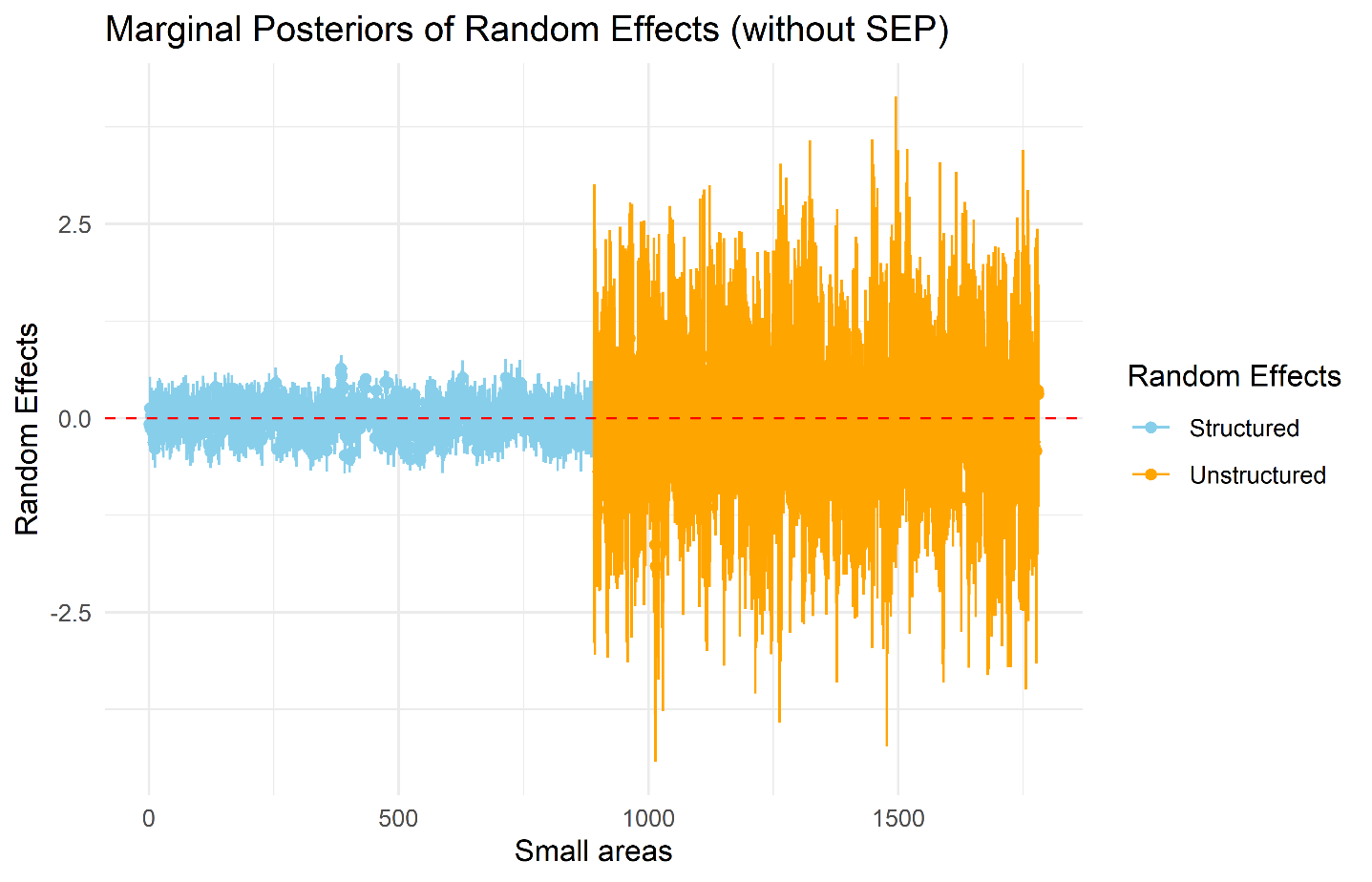


Figure S4. Marginal posteriors of random effects for small areas in the model with measures of SEP


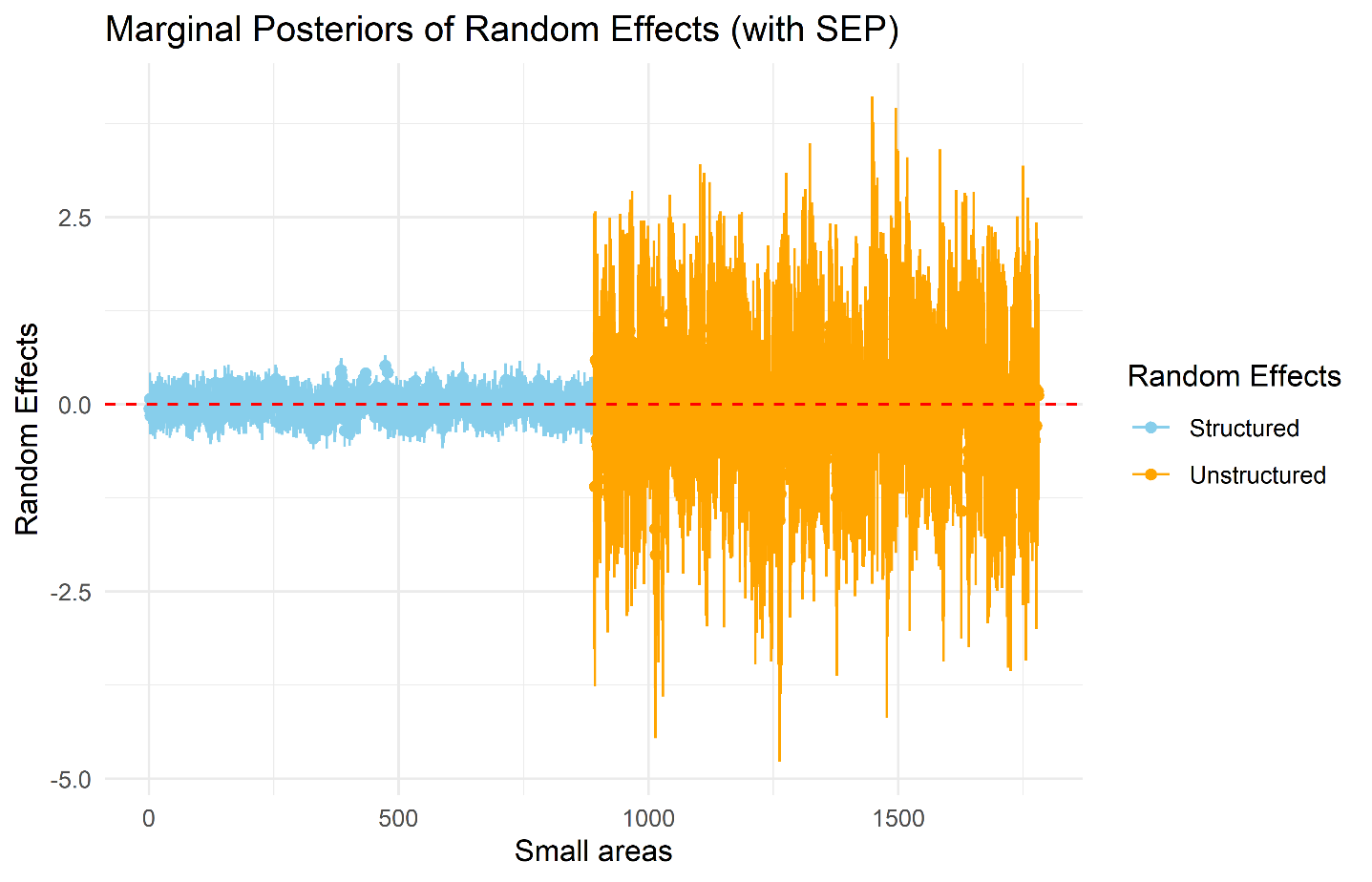


Figure S5. Marginal posteriors of random effects for municipalities in the model without any measures of SEP


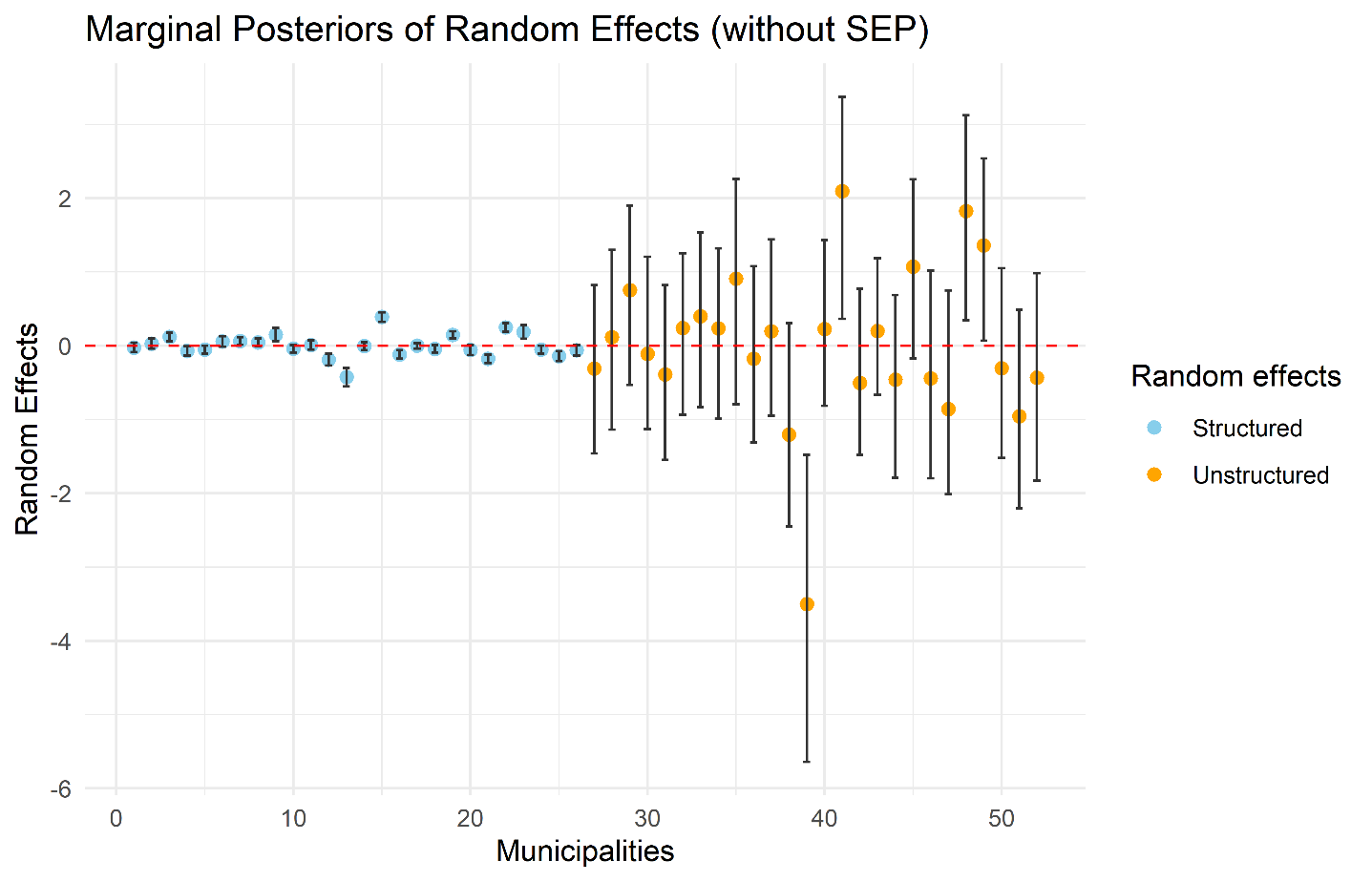


Figure S6. Marginal posteriors of random effects for municipalities in the model with measures of SEP


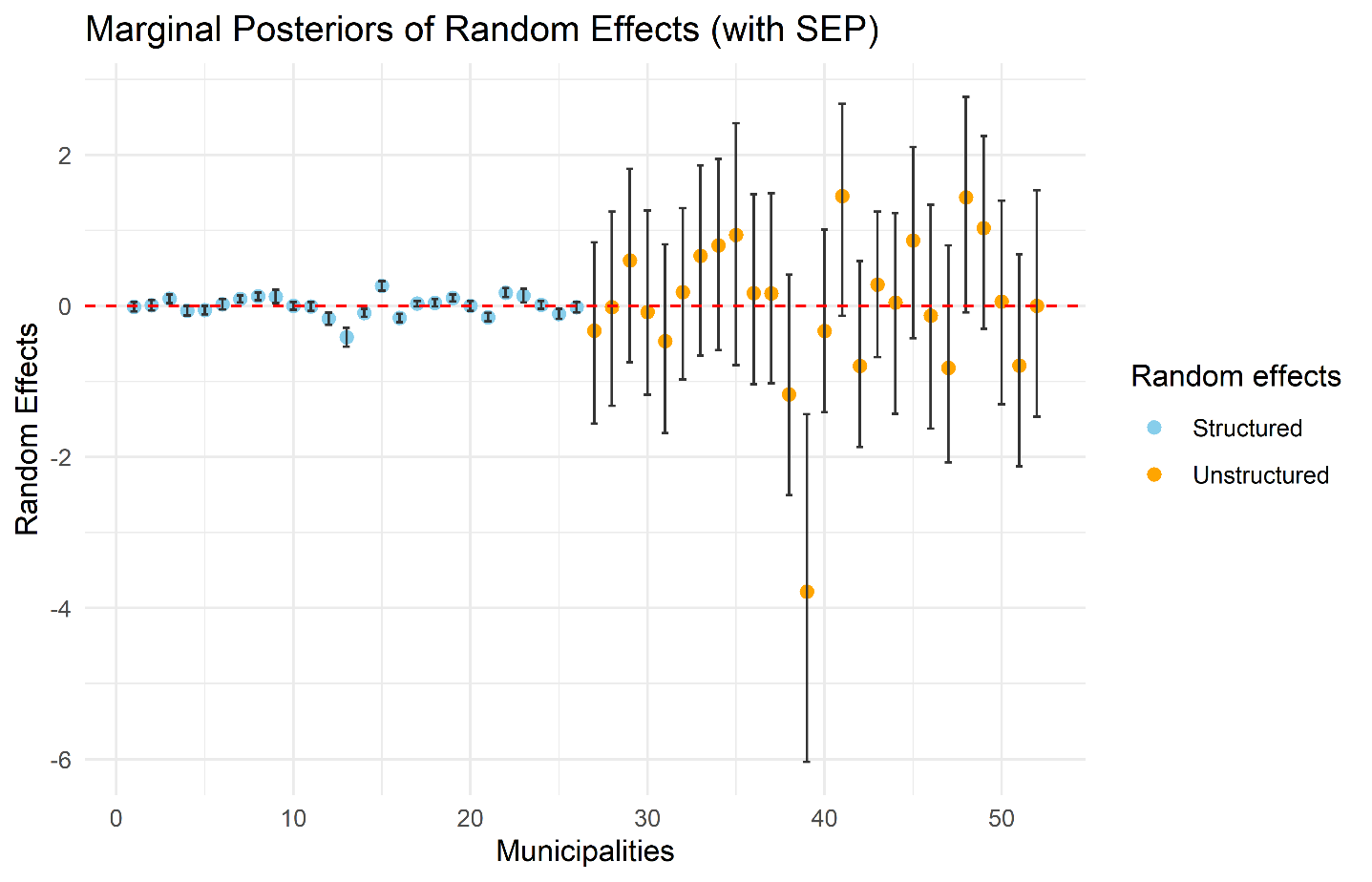


Figure S7. Density plot for the variance of random effects at the small area level


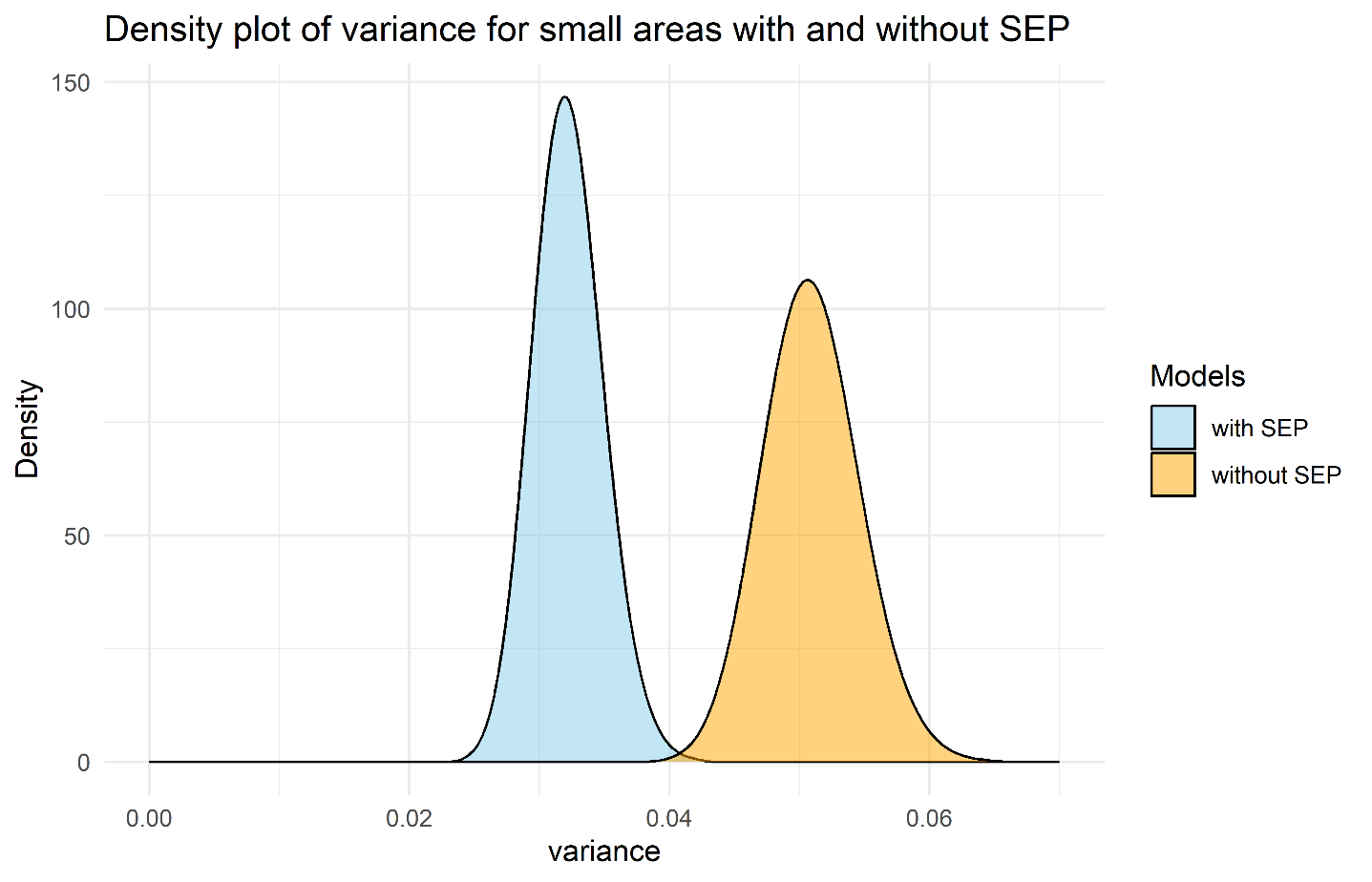


Figure S8. Density plot for the variance of random effects at the municipality level


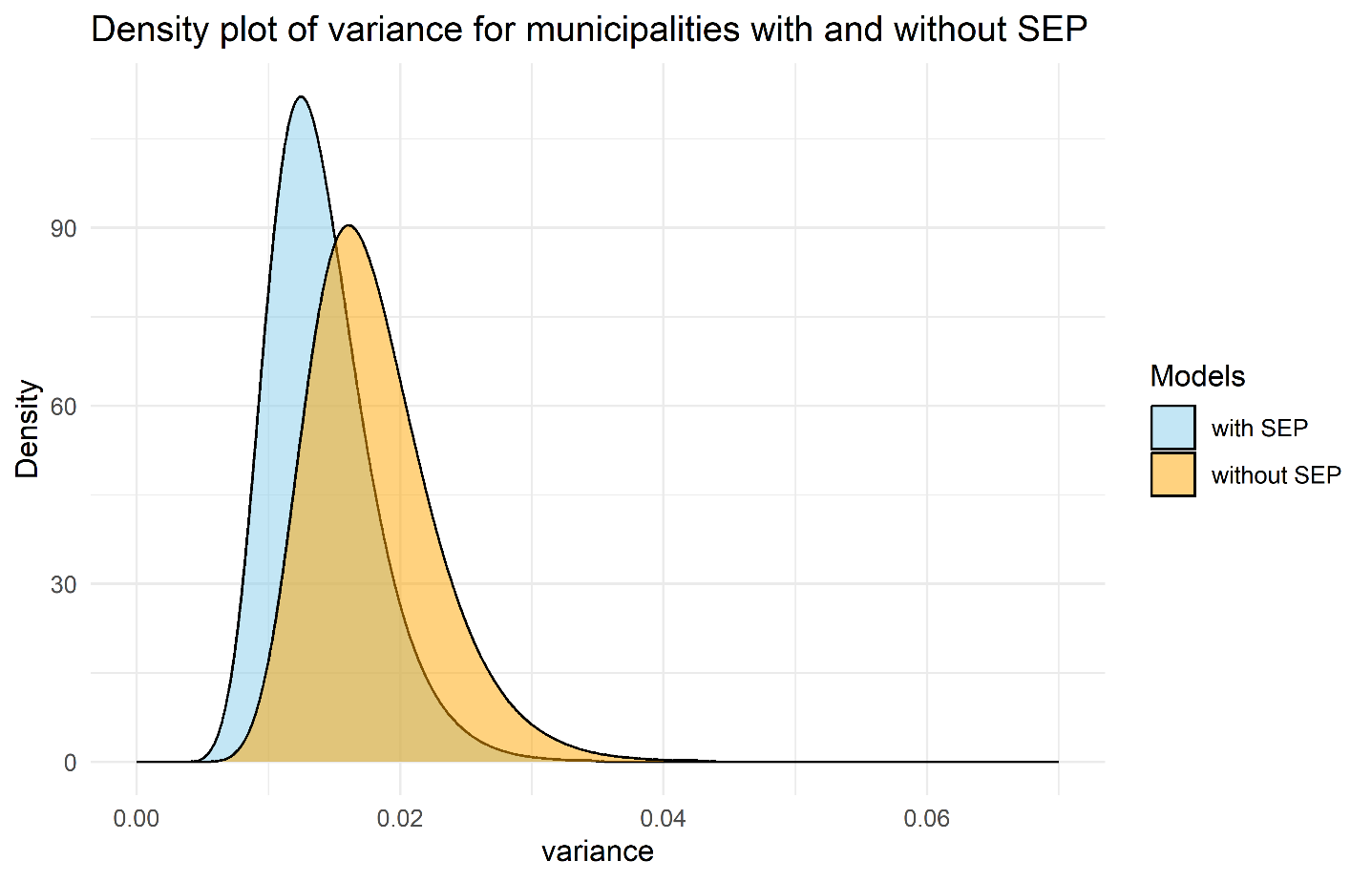


Figure S9. Density plot for the variance of random effects at the small area level by SEP measures at different levels

**
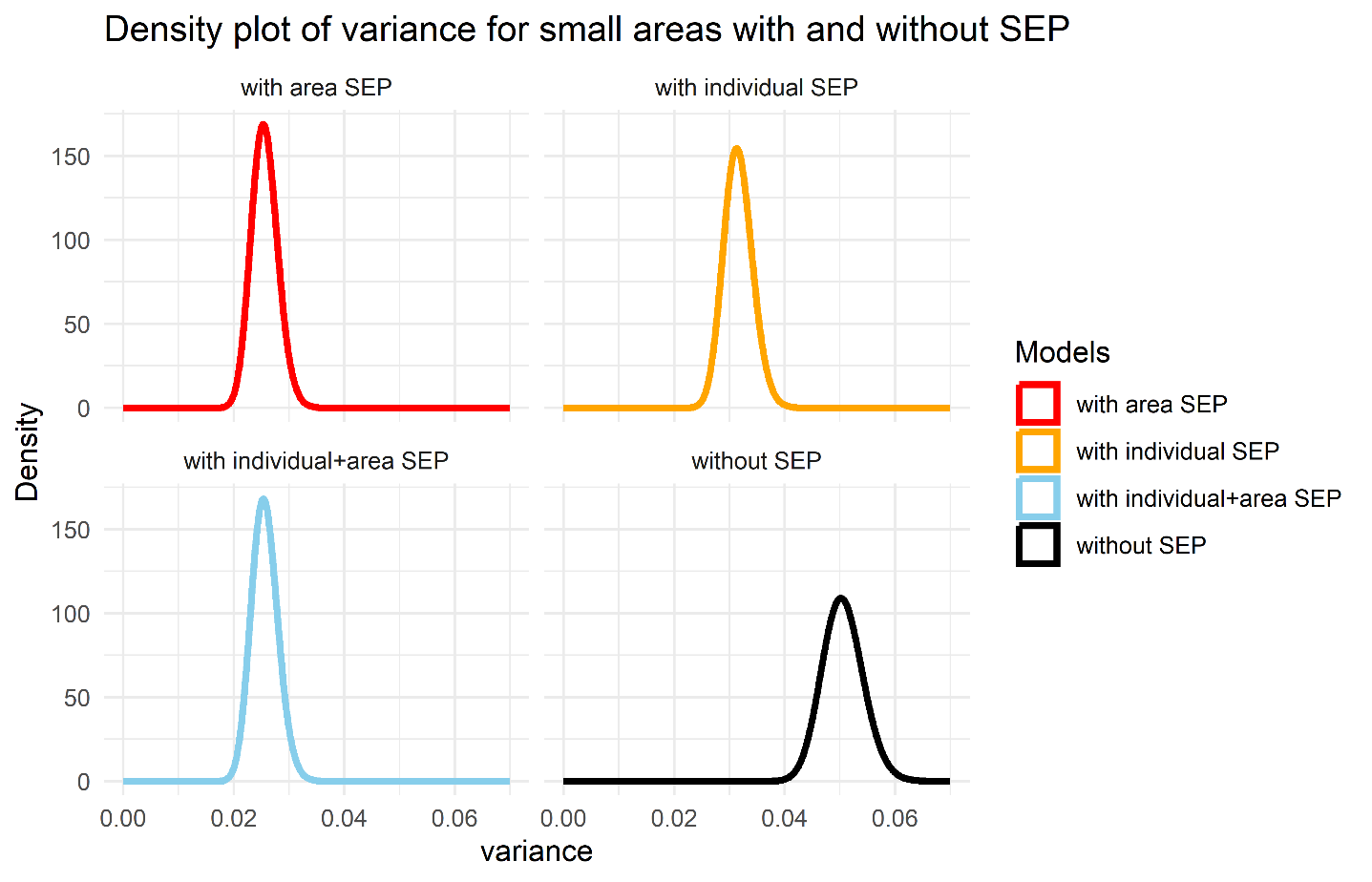
**
